# Monitoring Response Using Circulating Tumor DNA in Undifferentiated Pleomorphic Sarcoma: A Case Report

**DOI:** 10.7759/cureus.74837

**Published:** 2024-11-30

**Authors:** Amanda B Siy, Maggie Zhou, Ana Carolina A Boncompagni, Gregory Charville, George Poultsides, Kristen N Ganjoo

**Affiliations:** 1 Department of Medical Oncology, Stanford University, Stanford, USA; 2 Department of Hematology and Medical Oncology, Stanford University, Stanford, USA; 3 Department of Pathology, Stanford University, Stanford, USA; 4 Department of Surgical Oncology, Stanford University, Stanford, USA

**Keywords:** chemotherapy response, gemcitabine and docetaxel, magnetic resonance imaging, signatera ctdna, undifferentiated pleomorphic sarcoma

## Abstract

Circulating tumor DNA (ctDNA) can be used to assess treatment response in patients with undifferentiated pleomorphic sarcoma (UPS). The importance of this is explored in our case of a 75-year-old man who was diagnosed with UPS of the right kidney. After a right nephrectomy and tumor resection, the patient was recovering well with initially undetectable, and then slightly elevated, circulating tumor DNA. Abdominal pain started shortly before a scheduled magnetic resonance imaging (MRI) which revealed a large mass in the resection bed invading the liver. The patient was treated with gemcitabine and docetaxel chemotherapy, and the ctDNA level rose dramatically before gradually decreasing and eventually becoming undetectable. At surgery, pathologic examination of the re-resection specimen revealed a complete pathological response. ctDNA monitoring may be a useful tool for early detection of response to chemotherapy in patients with UPS.

## Introduction

Undifferentiated pleomorphic sarcoma, formally known as malignant fibrous histiocytoma, is a soft tissue sarcoma caused by abnormal fibroblast proliferation [1]. Histologically, these tumors are composed of a haphazard or storiform arrangement of highly pleomorphic or spindle-shaped cells, which have a variable amount of eosinophilic or amphophilic cytoplasm and numerous typical and atypical mitoses. It is most common in the extremities but can occur in the abdomen as well [2]. UPS commonly occurs in male patients between the ages of 60 and 85 [3]. Its etiopathogenesis is unclear, but UPS is known to develop in areas exposed to radiation and in scar tissue [4]. The mainstay of treatment for UPS is margin negative resection with or without radiation therapy; however, chemotherapy may be used for more advanced disease [5]. Previous studies have shown that docetaxel and gemcitabine is an effective treatment in patients with UPS [6]. Using circulating tumor DNA to monitor response was shown in this case and furthers research into the effectiveness of this tool; high levels of circulating tumor DNA (ctDNA) after the first dose of chemotherapy can indicate the tumor was very sensitive to chemotherapy leading to rapid cell death.

## Case presentation

**Figure 1:**
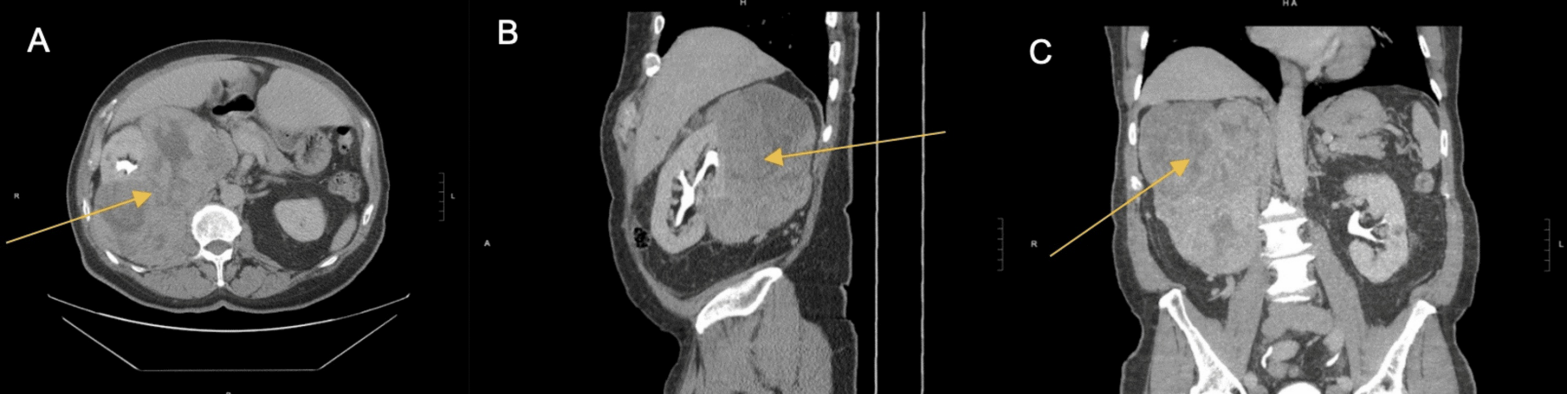
CT image. Orange arrows indicate evidence of mass measuring approximately 15.9 x 12.8 x 16.6 cm involving the right kidney. A, Axial. B, Sagittal, C, Coronal.

A 75-year-old man was found to have anemia when attempting to donate blood. He complained of gross hematuria and computed tomography (CT) on May 25, 2022, revealed a 16-cm right renal mass (Figures 1A-1C). Further work-up did not show metastatic disease. The patient underwent a right radical nephrectomy with en bloc resection of the tumor.

Surgery #1

Right radical nephrectomy was performed through an open laparotomy. The right renal mass was immediately identifiable due to the magnitude of its size, displacing the viscera into the left abdomen and pelvis. The vena cava was displaced medially and compressed cephalad to the tumor. Meticulous dissection of the renal vein and artery was indicated due to the extent of the parasitic tumor vasculature. The gonadal vein was divided near the confluence of the vena cava. The right adrenalectomy was also completed to ensure complete tumor resection. The patient suffered an ileus after discharge from the hospital and was treated medically. After the ileus resolved, the patient recovered without significant complications. The patient declined adjuvant therapy. 

Pathology #1

**Figure 2:**
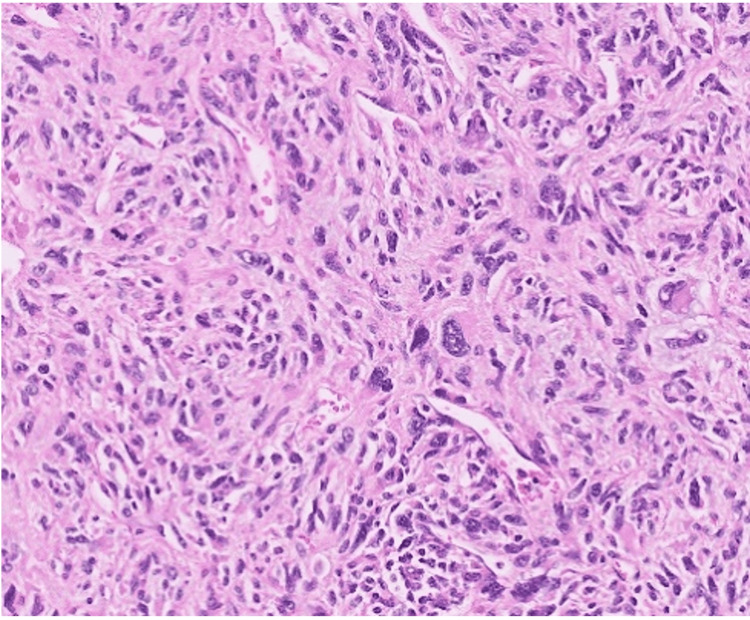
Representative photomicrograph at 400x magnification. On histological examination, the tumor consisted of a proliferation of markedly atypical spindle cells characterized by nuclear enlargement, pleomorphism, and hyperchromasia.

Pathology review showed a malignant, high-grade spindle and epithelioid neoplasm, best characterized as undifferentiated pleomorphic sarcoma with tumor involving the renal sinus and perinephric soft tissue (Figure 2). The tumor was 20 cm with negative surgical margins. Comprehensive genomic profiling (Altera, Natera, Inc) showed a CDK4 amplification and TP53 mutation. It also was negative for homozygous deletion of CDKN2A. There was increased mitotic activity, including atypical mitotic forms, and multifocal necrosis, accounting for less than 5% of the tumor volume prior to adjuvant therapy. Immunohistochemical stains were notable for heterogeneous, weak expression of CD34 and focal desmin immunoreactivity. There was no significant expression of p63, S100 protein, SOX10, PAX8, MDM2, STAT6, CK5, GATA3, myogenin, MyoD1, or SATB2. Expression of SDHB and SMARCB1 was retained. Fluorescence in situ hybridization (FISH) studies were negative for MDM2 gene amplification and ALK gene rearrangement.

Recurrence 

**Figure 3:**
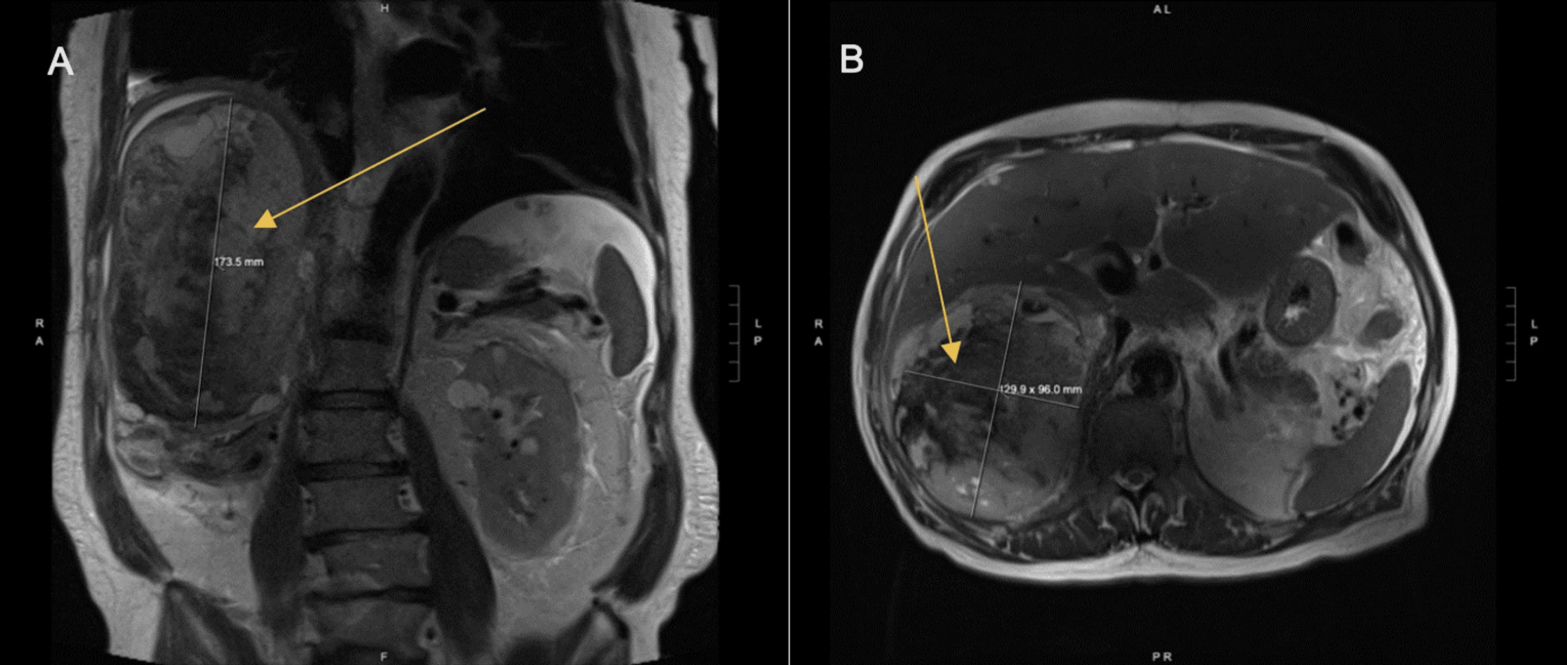
MRI image. Orange arrows indicate a 20 x 12.5 x 4 cm solid and cystic heterogeneously enhancing mass. A shows the coronal view of the mass. B shows the axial view of the mass.

The patient developed abdominal pain and bilateral lower extremity edema five months after the initial resection. Magnetic resonance imaging showed a new 20 x 12.5 x 4 cm, solid and cystic heterogeneously enhancing mass indenting/invading or arising from the posterior right hepatic lobe and extending into the right retroperitoneum from the diaphragm to the mid abdomen (Figure 3). The mass was causing severe external compression of the adjacent upper inferior vena cava which was likely causing the bilateral lower extremity edema. Then, two consecutive ctDNA measurements (Signatera, Natera, Inc) showed low (0.31 and 0.76 mean tumor molecules per milliliter) positive values (Figures 3A, 3B).

**Figure 4:**
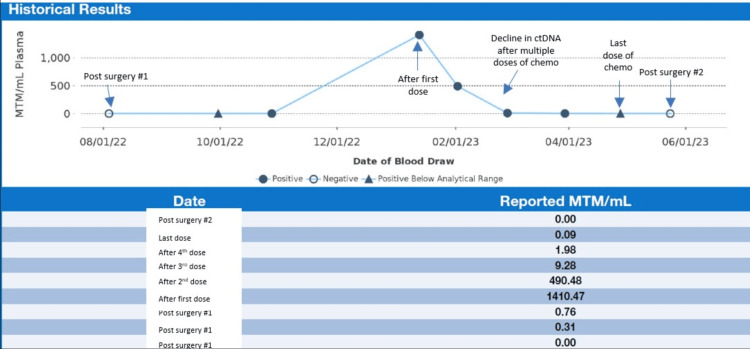
Signatera ctDNA results showing a peak in mean tumor molecules per milliliter after third dose of chemotherapy and then substantial decline after continuing chemotherapy. ctDNA: circulating tumor DNA, MTM: mean tumor molecule.

Given the rapid postoperative recurrence, after multidisciplinary discussion, the patient received systemic chemotherapy with gemcitabine (1,500 mg/m^2^) and docetaxel (50 mg/m^2^) every two weeks. The patient’s first ctDNA level after starting chemotherapy, measured after the second dose, was 1,410.47 mean tumor molecule (MTM)/mL, which rose from a baseline ctDNA level of near zero MTM/mL. This number was alarming, but we hypothesized that the tumor was so sensitive to chemotherapy that a majority of the tumor DNA cells spilled into the blood. After experiencing extreme pain in the area of the tumor after the first dose of chemotherapy, the patient reported clinical response to chemotherapy with decreasing abdominal pain, and the gemcitabine and docetaxel chemotherapy was continued. The patient’s ctDNA levels were measured every four to six weeks and decreased to 490.48 MTM/mL to 9.28, to 1.9 and finally to 0 MTM/mL (Figure 4). After nine doses of chemotherapy administered over 17 weeks, the patient showed good tolerance and continued tumor response was seen on MRI with a tumor size of 17.2 x 12.1x 10.4 cm. After two more doses, the tumor was further reduced in size to 16.0 x 11.6 x 9.8 cm. The rationale for giving 11 cycles of systemic therapy to downstage was due to evidence on the MRI showing continuous response and the tumor continuing to shrink. Myelosuppression was not an issue for this patient, and the patient also received growth factors. 

Surgery #2

**Figure 5:**
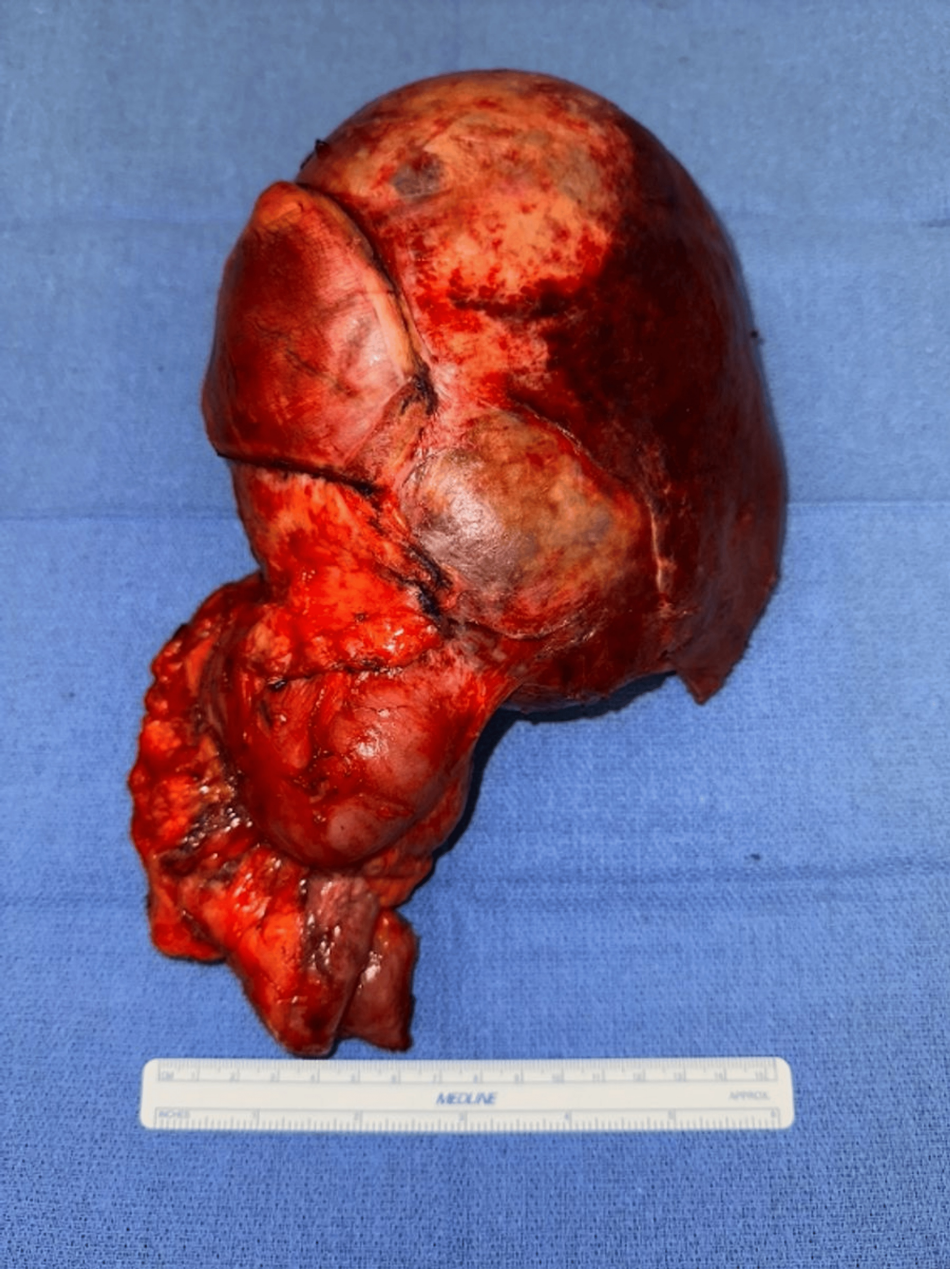
A 20 x 12.5 x 4 cm, solid and cystic heterogeneously enhancing mass invading the posterior right hepatic lobe and extending into the right retroperitoneum, right colon, and right hemi diaphragm.

The patient underwent surgical resection of the locally recurrent high-grade sarcoma. The same incision from the right radical nephrectomy was used, with some extension into the xiphoid and right costal margin. The small intestine was dissected off the right retroperitoneum and tumor, but the right colon was resected en bloc. A right posterior sectionectomy of the liver was performed en bloc after the tumor was identified to be invading the liver. En bloc resection of the right hemidiaphragm was also performed in order to achieve complete resection of the tumor, with the remaining diaphragmatic defect being repaired primarily. This allowed for the entire tumor to be removed with macroscopically negative surgical margins (Figure 5).

Pathology #2

**Figure 6:**
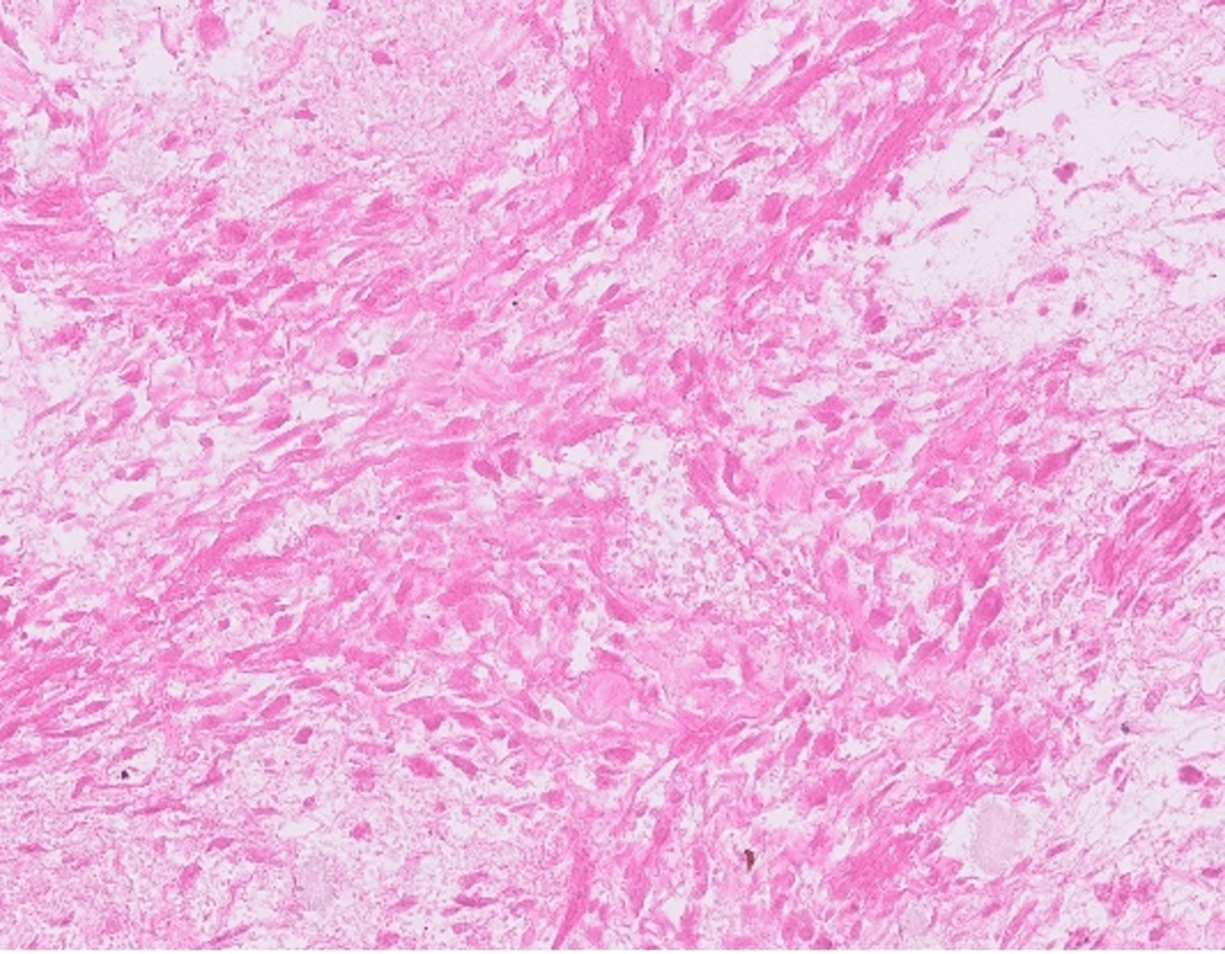
Representative photomicrograph at 400x magnification. Following adjuvant therapy, the tumor, which was extensively sampled for microscopic examination, was entirely necrotic.

Histological examination of the surgical specimen showed that the necrotic mass involved the liver and was adherent to the diaphragm and bowel serosa but did not invade the bowel wall. The bowel, liver, and diaphragmatic margins were negative, and the specimen was extensively sampled and showed no viable residual tumor (Figure 6). We continue to follow-up with this patient with positron emission tomography (PET) scans done every three months and ctDNA tests.

## Discussion

Circulating tumor DNA (ctDNA) refers to fragmented DNA present outside of cells derived from cancer cells. ctDNA is thought to be derived from various mechanisms, including tumor cell apoptosis, direct release from tumor cells, and secretion from macrophages following phagocytosis of tumor cells [7]. ctDNA has been postulated as a potential non-invasive biomarker to guide complex clinical decision-making, including assessing prognosis, detecting minimal residual disease after surgery, selecting high-risk patients for adjuvant therapy, tracking genetic changes, and monitoring response to systemic therapy. While ctDNA is increasingly validated in other solid tumors like colon cancer and lymphoma [8,9], there are unique challenges to its application in soft tissue sarcoma.

First and foremost, the rarity of sarcomas makes it difficult to access sufficiently large enough datasets to test and validate ctDNA platforms. Moreover, the choice of methodology for ctDNA detection requires a sub-type specific approach due to the heterogeneity of sarcoma genomics. For instance, unlike carcinomas, recurrent gain-of-function single nucleotide variants (SNVs) are rare in sarcomas. With the notable exception of gastrointestinal stromal tumor which is predominantly driven by gain-of-function mutations in the genes encoding KIT or PDGFRA receptor tyrosine kinases [10,11], it is challenging to design a ctDNA assay in sarcoma dependent on recurrent SNVs. Sarcomas are more commonly characterized by loss-of-function mutations of tumor suppressor genes, recurrent patterns of structural variants like copy number alterations and translocations, aneuploidy, and alteration of genome-wide methylation patterns [12]. Finally, data suggest lower shedding of tumor material in sarcomas compared to other tumors of similar size [13].

Despite these challenges, there is increasing interest in the application of ctDNA technology to sarcoma due to the high unmet need in this disease. Recent studies have demonstrated feasibility of ctDNA quantification in Ewing sarcoma, osteosarcoma, rhabdomyosarcoma, and synovial sarcoma [14,15,16]. ctDNA variant allele fraction was also demonstrated to be correlated with disease response and survival in a phase 2 trial combining ipilimumab and nivolumab with cryoablation [17].

In our case report, we demonstrate proof of concept in using the Signatera™ ctDNA measurable residual disease (MRD) assay to assess disease response to chemotherapy in undifferentiated pleomorphic sarcoma. As described previously, Signatera™ ctDNA MRD assay is a personalized, tumor-informed 16-plex polymerase chain reaction next-generation sequencing assay used for the detection and quantification of ctDNA in blood samples [18]. Signatera has previously demonstrated feasibility in assessing disease kinetics in in leiomyosarcoma [19] and also in detecting residual disease in leiomyosarcoma, undifferentiated pleomorphic sarcoma, and dedifferentiated liposarcoma [20]. Our case report contributes to a growing body of evidence describing the utility of ctDNA strategies and its interpretation in sarcoma.

## Conclusions

ctDNA is an emerging tool for cancer patients to assist in molecular and genomic analysis, detect minimal residual disease after locoregional intervention, improve risk stratification, and tailor systemic therapy. Our case of its application in assessing response to chemotherapy in a 75-year-old male with undifferentiated pleomorphic sarcoma adds to a growing body of evidence of its utility in sarcoma. After a nephrectomy with negative margins, our patient experienced disease recurrence in the posterior right liver. We utilized Signatera ctDNA levels to track his response to the docetaxel and gemcitabine chemotherapy regimen. We continued the regimen despite a significant rise in ctDNA levels after the first two doses of therapy as we hypothesized the sharp rise was a result of initial tumor lysis and indicated response to therapy. This proved to be an accurate prediction and an effective course of treatment as the final resection of the mass showed no viable residual tumor. Our experience supports the utility of ctDNA monitoring as a non-invasive biomarker for assessing response to treatment. Prospective studies are needed to validate its use in larger datasets and across other sarcoma histological subtypes.
